# UV-Assisted Silver Functionalization of Cotton Gauze for Antimicrobial and Biocompatible Wound Healing Applications

**DOI:** 10.3390/microorganisms14010213

**Published:** 2026-01-16

**Authors:** Rebecca Pellegrino, Carmen Lanzillotti, Mauro Pollini, Federica Paladini

**Affiliations:** 1 Department of Engineering for Innovation, University of Salento, 73100 Lecce, Italy; rebecca.pellegrino@unisalento.it; 2 Caresilk S.r.l.s., c/o Dhitech, Via Monteroni, 73100 Lecce, Italy; carmen.lanzillotti@caresilk.it; 3 Department of Experimental Medicine, University of Salento, 73100 Lecce, Italy; mauro.pollini@unisalento.it

**Keywords:** wound dressing, silver nanoparticles, UV-assisted deposition method, antimicrobial, wound healing

## Abstract

Bacterial infections remain a major challenge to human health, especially in wound healing, where they can cause prolonged inflammation, delayed recovery, and severe complications. Current research is increasingly focused on developing innovative antimicrobial materials capable of overcoming the limitations of conventional antibiotics, whose effectiveness has declined due to the rise in bacterial resistance. Among the various alternatives, silver nanoparticles have gained particular attention for their broad-spectrum antibacterial properties and have already been successfully applied in the functionalization of commercial wound dressings. The aim of this study was to optimize the functionalization of commercial cotton gauzes based on in situ UV-assisted reduction of silver nanoparticles, reducing methanol usage and identifying the minimal silver nitrate precursor concentration to achieve antimicrobial efficacy while maintaining biocompatibility. Different precursor concentrations were then evaluated through cytocompatibility assays (MTT, Live/Dead, and scratch tests on fibroblasts) and antimicrobial analyses against *Escherichia coli*, *Pseudomonas aeruginosa*, *Staphylococcus aureus* (including an antibiotic-resistant strain), and *Candida albicans*. The results demonstrated that a 0.5% *w*/*w* silver nitrate concentration provided strong antimicrobial and antibiofilm activity without compromising textile properties or cytocompatibility. Furthermore, this optimized process reduced material waste, highlighting its potential for scalable production of antimicrobial wound dressings.

## 1. Introduction

Bacterial infections remain a major challenge to human health. In particular, during wound healing, open wounds are easily contaminated by bacteria originating from the surrounding environment, the patient’s own skin, or endogenous microflora [1,2,3,4]. Once infection occurs, it creates a hostile microenvironment that makes healing more difficult. This can complicate treatment, prolong inflammation, and delay patient recovery, and may lead to severe local or systemic complications [5,6]. Therefore, the timely and appropriate use of antibacterial agents plays a vital role in promoting wound repair.

For decades, antibiotics have served as the primary strategy for treating bacterial infections. However, their systemic use and nonspecific action have contributed to the emergence of bacterial resistance. As a result, antibiotics are becoming less effective, and many patients no longer respond to them [7,8,9]. To address these limitations, modern wound dressings often incorporate innovative antimicrobial components. These materials can combat bacterial proliferation while keeping the wound moist, allowing gas exchange and wound exudate absorption, as well as promoting cell migration and proliferation [10,11]. Nanotechnology has emerged as a promising tool for developing such materials [12]. Many nanomaterials have been studied, such as carbon-based, metallic, and metal oxide systems, with unique physicochemical properties [13]. Among them, silver nanoparticles (AgNPs) are currently the most widely used in wound care [14]. Their high surface-to-volume ratio enhances interactions with biological systems and gives them strong antimicrobial efficacy. The antibacterial mechanism of AgNPs involves several pathways: (i) the release of silver ions that interact with bacterial enzymes, (ii) the generation of Reactive Oxygen Species (ROS) that cause oxidative stress, (iii) the disruption of cell membranes, (iv) interference with metabolic processes, and (v) damage to bacterial DNA [15,16]. They have also demonstrated broad-spectrum activity against both Gram-positive and Gram-negative bacteria, including antibiotic-resistant strains. They are therefore very promising for biomedical applications, particularly in wound dressings [17,18]. Several studies have explored this approach with encouraging results. Notably, the method proposed by Pollini et al. is of particular significance due to its simplicity, scalability, and high performance in achieving antimicrobial functionality [19,20]. Compared with conventional chemical methods, the process is more convenient in terms of both time and cost, as it does not require binders, complex reducing agents, or expensive equipment [21].

The goal of the present work was to optimize this in situ UV-assisted deposition of AgNPs for the functionalization of commercial cotton gauzes. The study aimed to minimize methanol content and determine the lowest effective concentration of silver nitrate (AgNO_3_) precursor to achieve potent antimicrobial activity and biocompatibility, while reducing material waste. To this end, different AgNO_3_ concentrations were tested and compared in terms of material loss during processing, cytocompatibility (via MTT, Live/Dead, and scratch assays on fibroblasts), and antimicrobial performance (through microbial growth inhibition and antibiofilm assays). Antimicrobial activity was evaluated against *Escherichia coli* ATCC 25922 and *Pseudomonas aeruginosa* ATCC 27853 (Gram-negative), *Staphylococcus aureus* ATCC 29213 and ATCC 43300 (Gram-positive, the latter antibiotic-resistant), and the fungus *Candida albicans* ATCC 14053. Commercial cotton gauzes were selected as the substrate in this work since they are widely used in wound care applications, and enhancing an existing, clinically adopted product may offer substantial practical and commercial benefits. Additional analyses were performed to evaluate potential alterations in the intrinsic properties of commercial gauzes.

## 2. Materials and Methods

### 2.1. Materials

Silver nitrate (AgNO_3_, 99+%) was purchased from Alfa Aesar (Fisher Scientific, Waltham, MA, USA); methanol (≥99.9%, MW 32.04), sodium chloride (NaCl, ≥99%, MW 58.44), Phosphate-Buffered Saline tablets (PBS), calcium chloride dihydrate (CaCl_2_·2H_2_O, ≥99%, MW 147.01), sodium bicarbonate (NaHCO_3_, ≥99%, MW 84.01), potassium chloride (KCl, MW 74.55), and albumin bovine serum (BSA, for microbial culture) were purchased from Sigma Aldrich (Saint Louis, MO, USA); sterile cotton gauzes (10 × 10 cm^2^) were purchased from a local pharmacy. All aqueous solutions were prepared with distilled water.

### 2.2. Treatment of Cotton Gauzes

Commercially available sterile cotton gauzes were functionalized with AgNPs via in situ photo-reduction of AgNO_3_, following the procedure described in a previous work [20]. In this study, the concentration of methanol used as a reducing agent in the silver-based solution was fixed at 5% *w*/*v*, while four different AgNO_3_ concentrations were tested: 0.1% *w*/*w*, 0.5% *w*/*w*, 2% *w*/*w*, and 4% *w*/*w*. For each condition, the solution was used to impregnate the gauze by dip-coating for 5 min at room temperature (RT). Samples were then exposed to ultraviolet (UV) irradiation (365 nm, 500 W, 20 cm distance) for 15 min on each side, washed three times with distilled water, and dried at RT. The samples were designed as follows: untreated gauze (CTRL), and gauze treated with 0.1% AgNO_3_ (T01), with 0.5% AgNO_3_ (T05), with 2% AgNO_3_ (T2), and with 4% AgNO_3_ (T4).

### 2.3. Quantification of Ag^+^

The concentration of silver ions (Ag^+^) was determined using a colorimetric assay based on the reaction between Ag^+^_(aq)_ and chloride ions (Cl^−^_(aq)_), which produces a colloidal silver chloride suspension with a silvery white color detectable spectrophotometrically.

Firstly, a calibration curve was generated by preparing a series of AgNO_3_ standard solutions at known concentrations. Each standard solution was mixed 1:1 (*v*/*v*) with a 0.1 M NaCl solution, and after 10 min the absorbance of the resulting suspensions was measured at 450 nm using a Jasco V-660 UV–visible spectrophotometer (Jasco, Palo Alto, CA, USA). The calibration curve was constructed by plotting absorbance versus Ag^+^ concentration.

The same procedure was applied to the experimental samples, namely the precursor solution (before and after gauze impregnation) and the distilled water collected after the first washing step. When the measured absorbance exceeded the calibration range, the samples were diluted appropriately with distilled water prior to analysis. The corresponding Ag^+^ concentration was then calculated by interpolating the diluted sample value on the calibration curve and multiplying by the respective dilution factors.

It was therefore possible to calculate the amount of reacted Ag^+^ according to Equation (1):

(1) $$Reacted\;\left[{Ag}^{+}\right]\left(ppm\right)=\left({Ag}_{before}-{Ag}_{after}\right)-{Ag}_{unreacted},$$

where Ag_unreacted_ is the amount of Ag^+^ in the distilled water after the first washing step, and Ag_before_ and Ag_after_ are the amount of Ag^+^ in the precursor solution before and after gauze impregnation, respectively.

### 2.4. Add-On Ratio

The add-on ratio of the treated samples (T01, T05, T2, and T4) was determined by weighing the specimens before and after treatment using an analytical balance (Kern ABJ, Kern & Sohn GmbH, Balingen, Germany; readability = 0.1 mg). The add-on ratio was calculated according to Equation (2):

(2) $$Add-on\;\left(\%\right)=\frac{w_{f}-w_{i}}{w_{i}}\times 100,$$

where w_f_ is the dry weight after the treatment, and w_i_ is the initial dry weight of the sample before the treatment. An average of four samples were recorded [22].

### 2.5. Absorption and Retention of Fluids

As a wound dressing material, a key property of cotton gauze is its ability to absorb and retain liquids. Absorption and retention tests were therefore performed using three different fluids: distilled water used as a reference, simulated wound fluid (SWF, composition: 5.844 g NaCl, 3.360 g NaHCO_3_, 0.298 g KCl, 0.278 g CaCl_2_, 33 g BSA, 1000 g distilled water [23]), and defibrinated bovine blood (Biotec, Grosseto, Italy).

For the absorption test, 3 × 3 cm^2^ specimens (CTRL, T01, T05, T2, T4) were weighed and immersed in 10 mL of each test fluid for 30 min at ambient temperature (26 ± 2 °C). Samples were then removed, gently held for 10 s to allow excess liquid to drain, and immediately weighed to determine the wet mass. Absorption was calculated according to Equation (3):

(3) $$A\;\left(\%\right)=\frac{w_{f}-w_{i}}{w_{i}}\times 100,$$

where A is the absorption percentage, w_f_ the wet mass, and w_i_ the initial dry mass [24].

Retention capacity was evaluated on the same specimens obtained from the absorption test. After absorption measurements, samples were left to dry under ambient conditions (26 ± 2 °C) and weighed every 15 min until they reached their initial dry weight. Retention was expressed as a relative percentage, defined as the ratio between the residual liquid retained in the sample and the initially absorbed liquid, according to Equation (4):

(4) $$R\;\left(\%\right)=\left(1-\left|\frac{w_{t}-w_{i}}{w_{0}-w_{i}}\right|\right)\times 100,$$

where R is the retention percentage at time t, w_t_ is the sample weight at time t, w_i_ is the initial dry weight, and w_0_ is the wet weight immediately after absorption. By definition, retention equals 100% at t = 0 (immediately after fluid uptake) and decreases to 0% when the sample reaches its initial dry weight. The time point at which R = 0% is defined as the retention time and reported in this work.

These two tests were performed in triplicate for each sample.

### 2.6. Vertical Wicking Test

Vertical wicking was evaluated using distilled water. Rectangular specimens (90 × 10 mm^2^) of each sample type (CTRL, T01, T05, T2, T4) were suspended vertically with their lower edge immersed in approximately 3 mm of distilled water contained in a Petri dish. To facilitate the visualization of the capillary rise front, a diluted Crystal Violet (CV) solution (10% *v*/*v*) was added as a chromatic tracer. The height of liquid uptake along the sample was recorded after 1, 5, and 10 min. The test was performed in triplicate, and the mean values were reported [24].

### 2.7. Antimicrobial Properties of Untreated and Treated Cotton Gauzes

The antimicrobial activity of untreated (CTRL) and treated (T01, T05, T2, and T4) gauzes was evaluated against the reference strains *Escherichia coli* (*E. coli*, ATCC 25922), *Pseudomonas aeruginosa* (*P. aeruginosa*, ATCC 27853), and *Staphylococcus aureus* (*S. aureus*, ATCC 29213); methicillin-resistant (MRSA) *Staphylococcus aureus* ATCC 43300; and the fungal reference strain *Candida albicans* (*C. albicans*, ATCC 14053). Before performing each test, a single colony of each tested microorganism was first cultured in Tryptic Soy Broth (TSB) for bacteria or Potato Dextrose Broth (PDB) for fungi at 37 °C for 18 h.

#### 2.7.1. Qualitative Antimicrobial Properties of Cotton Gauzes

The antimicrobial activity of untreated and treated gauzes (CTRL, T01, T05, T2, and T4) was qualitatively evaluated using the agar diffusion method, by measuring the zones of bacterial growth inhibition around the samples. Cotton gauze samples (1 × 1 cm^2^) were incubated at 37 °C for 18 h in contact with each microbial species on nutrient agar plates, and then the presence of an area of inhibited bacteria growth was evaluated around them. The width of the inhibition zones surrounding the samples was measured in the two directions around them using ImageJ software (version 1.54c14, National Institutes of Health, Bethesda, MD, USA), and the mean values ± S.D. were reported. The antimicrobial effectiveness of the treated and untreated gauzes was determined according to the levels provided by the standard ‘SNV 195920-1992’ [25]. Thus, if the width of the inhibition zone was larger than 1 mm, “good” antibacterial activity was associated with the sample; on the other hand, if the sample was fully covered by bacteria, its antibacterial activity was labeled as “insufficient”.

#### 2.7.2. Microbial Growth Curves in the Presence of Untreated and Treated Cotton Gauzes and Antimicrobial Efficacy (AME)

To evaluate microbial growth inhibition, a portion of each pre-cultured microorganism was diluted in fresh culture medium to a final volume of 10 mL, with an OD_600_ value of 0.0005. The resulting microbial suspensions were incubated with 2 × 1 cm^2^ gauze samples at 37 °C under static conditions. At pre-determined time intervals (2, 4, 6, and 8 h) 1 mL of the growth medium was taken, and the OD_600_ was measured using a UV-Vis spectrophotometer (V-1200, VWR, Radnor, PA, USA). The percentage of antimicrobial efficacy (AME) of the treated samples was calculated relative to the untreated control (CTRL) according to Equation (5):

(5) $$AME\;\left(\%\right)=\left|\frac{N_{T}-N_{CTRL}}{N_{CTRL}}\right|\times 100,$$

where N_T_ and N_CTRL_ are the OD_600_ values in the presence of the treated (T01, T05, T2, and T4) and untreated (CTRL) samples, respectively.

This test was performed in duplicate for each sample.

#### 2.7.3. Biofilm Formation in the Presence of Untreated and Treated Gauzes

To evaluate biofilm formation, samples of untreated (CTRL) and treated (T01, T05, T2, and T4) cotton gauzes (1 × 1 cm^2^) were placed in a 24-well microtiter and incubated for 4 h and 24 h at 37 °C with 2 mL of nutrient broth inoculated with each microbial suspension (initial cell density 1 × 10^6^ CFU/mL). After incubation, the gauze samples and media were removed from the wells, and the surfaces were gently washed with PBS to remove non-adherent microorganisms. Then, 1 mL of 0.1% *v*/*v* CV aqueous solution was added to each well and kept in the dark for 15 min to stain the adherent biofilm. The CV solution was then discarded, and the wells were rinsed three times with PBS to remove non-absorbed dye, obtaining stained specimens corresponding to the amount of biofilm formed. Finally, 200 µL of 30% *v*/*v* glacial acetic acid was added to each well and left for 15 min to extract the bound dye. The absorbance of the resulting solution was measured at 590 nm using an EnVision Multimode Plate Reader (PerkinElmer, Waltham, MA, USA). The biofilm reduction (BR) was calculated relative to the untreated control (CTRL) using Equation (6):

(6) $$BR\;\left(\%\right)=\left|\frac{B_{T}-B_{CTRL}}{B_{CTRL}}\right|\times 100,$$

where B_T_ and B_CTRL_ are the OD_590_ values in the presence of the treated (T01, T05, T2, and T4) and untreated (CTRL) samples, respectively.

This test was performed in duplicate for each sample.

### 2.8. Biocompatibility Assessment

The biocompatibility of gauze in a dry state, either untreated (CTRL) or functionalized with different silver concentrations (T01, T05, T2, and T4), was assessed using 3T3 murine fibroblasts. Cells were cultured in Dulbecco’s Modified Eagle Medium (DMEM; Sigma Aldrich), supplemented with 10% fetal bovine serum (FBS), 1% antibiotics (100 U/mL penicillin and 100 mg/mL streptomycin), and 2 mM L-glutamine. Cell cultures were maintained at 37 °C in a humidified incubator (Heracell, Thermo Scientific, Waltham, MA, USA) under 5% CO_2_, with medium replacement every three days. For the experimental setup, fibroblasts were seeded at a density of 1.5 × 10^4^ cells per well in contact with CTRL, T01, T05, T2, and T4, while tissue culture polystyrene (TCPS) served as the control condition.

#### 2.8.1. MTT Assay

The viability of cells in contact with CTRL, T01, T05, T2, and T4 was quantified by performing an MTT assay [3-(4,5-dimethylthiazol-2-yl)-2,5-diphenyltetrazolium bromide; Sigma Aldrich]. 3T3 fibroblasts were cultured directly on the samples and on TCPS, used as the control substrate. The assay was performed in triplicate for each condition at 1, 2, and 3 days of cell culture. MTT solution (5 mg/mL in PBS) was diluted in fresh medium to a final concentration of 0.5 mg/mL and added to each well. Plates were incubated at 37 °C for 3 h, after which the resulting formazan crystals were solubilized with Dimethyl Sulfoxide (DMSO). Absorbance was recorded at 540 nm using a Multimode Plate Reader EnVision (PerkinElmer, Waltham, MA, USA) [26].

#### 2.8.2. Live/Dead Assay

A Live/Dead assay was performed on 3T3 fibroblast cultures grown directly in contact with the CTRL, T01, T05, T2, and T4 samples, as well as with TCPS, used as a control, to qualitatively assess the biocompatibility of the samples. The assay was conducted after 3 days of culture. Cells grown directly on coverslips, previously placed on the bottom of the culture plate, were incubated for 15 min at 37 °C with a staining solution containing 2 μmol/L calcein-AM (acetomethoxy derivative of calcein) and 2 μmol/L propidium iodide in PBS. Following staining, live and dead cells were visualized using a fluorescence microscope (Axio Vert A1, Zeiss, Oberkochen, Germany) at 20× magnification, and images were analyzed with AxioVision software (Zeiss ZEN 3.11) [27].

#### 2.8.3. In Vitro Scratch Assay

A scratch assay, involving the disruption of a confluent cell monolayer, was performed on 3T3 fibroblasts cultured in contact with CTRL, T01, T05, T2, and T4, with TCPS serving as the control, to evaluate the wound healing potential of the gauzes. Fibroblasts were seeded in 24-well plates at a density of 1.5 × 10^4^ cells/mL and maintained until confluence. A straight scratch (~1 mm in width) was created across the monolayer using a sterile pipette tip. Following PBS washing to remove detached cells and debris, cultures were maintained in the presence of gauzes. Wound closure was monitored by acquiring images at 0 and 3 days with an optical microscope (Axio Vert A1, Zeiss). The medium was replaced at Time 0 and Time 2 according to the cell culture setup.

### 2.9. Statistical Analysis

The data are presented as the mean ± Standard Deviation (S.D.) for the indicated number of experiments. The statistical analysis was conducted by using One- and Two-way ANOVA. In all comparisons, *p* < 0.05 was considered statistically significant, and the *p*-values are reported for statistically significant results. All ANOVA post hoc analyses were performed using Tukey’s test.

## 3. Results

### 3.1. Treatment and Characterization of Cotton Gauzes

To enhance the antimicrobial performance of a commercial wound dressing, cotton gauzes were functionalized through a simple and scalable dip-coating process using aqueous AgNO_3_ solutions, followed by UV irradiation to photo-reduce Ag^+^ ions into AgNPs. The precursor solutions, both before and after the treatment of a single sample, as well as the distilled water collected after the first washing step, were analyzed by UV-Vis spectroscopy to quantify the amount of unreacted Ag^+^ remaining at each concentration, using the calibration curve obtained from AgNO_3_ standards (A = 0.0023 C + 0.0017, R^2^ = 0.992; where A is the absorbance and C the Ag^+^ concentration in ppm). As shown in Table 1, the amount of reacted Ag^+^ increased with the Ag^+^ concentration in the precursor solution, as expected. However, an increase in the amount of unreacted Ag^+^ was also observed, exceeding the reacted fraction for samples T2 and T4, indicating a significant loss of silver due to incomplete reaction and subsequent removal during the washing step.

**Figure 1:**
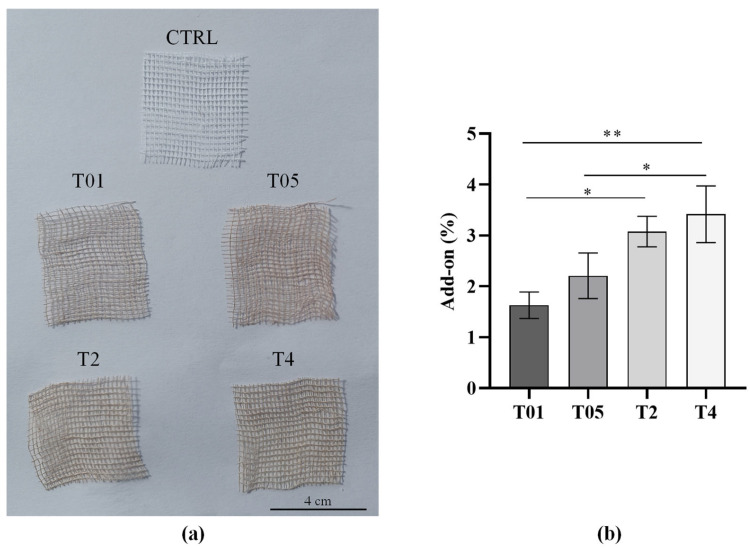
(**a**) Untreated (CTRL) and treated (T01, T05, T2, T4) samples; (**b**) Add-on ratios. * *p* < 0.05; ** *p* < 0.01.

The increase in reacted Ag^+^ was further confirmed by the add-on ratio, which estimates the amount of AgNPs formed on the treated gauzes after the UV irradiation and washing steps. As shown in Figure 1b, the add-on percentage increased with the Ag^+^ concentration in the precursor solution. Moreover, a distinct color change from white to brown was observed with the naked eye after treatment at all concentrations, consistent with the formation of AgNPs on the gauze surface. In fact, cotton gauzes became darker in color as the content of AgNPs increased compared with CTRL group (Figure 1a).

**Figure 2:**
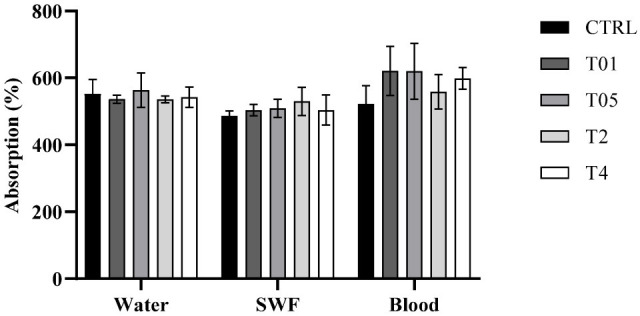
Absorption of fluids (water, SWF, and blood).

To evaluate whether AgNP functionalization affected the intrinsic properties of the cotton gauze, various tests were performed. In particular, the ability to absorb and retain fluids was investigated, as this represents a critical parameter for wound dressing applications. Distilled water was used as a reference fluid, while SWF was prepared to mimic the composition of wound exudate. Since the purpose of this study was not to investigate coagulation mechanisms, standardized defibrinated bovine blood was selected to eliminate clotting effects that could interfere with data consistency, allowing us to obtain reproducible measurements exclusively on material interaction with blood components under controlled conditions. As shown in Figure 2, the gauzes exhibited a high absorption capacity for all tested fluids, with a weight increase exceeding 550% relative to the initial dry mass in all cases. It was evident that the silver treatment did not impair the performance of the material, as no significant differences were observed among the different sample groups for any of the tested fluids.

Regarding fluid retention time, all samples showed similar behavior in SWF and water, as reported in Table 2. Some differences emerged in the case of blood, with higher retention time observed for samples T01 and T05.

**Figure 3:**
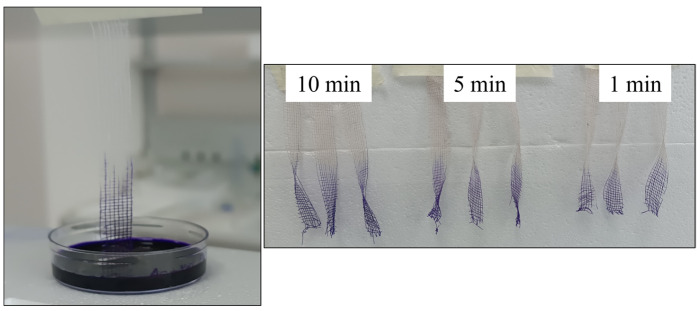
Representative image of vertical wicking test setup.

To evaluate the vertical wicking ability, the samples were tested at 1, 5, and 10 min, and each measurement was performed in triplicate; the mean values are reported in Table 3, while a representative image of the testing setup is reported in Figure 3. The results showed no statistically significant differences among the samples, except at the 1 min time point. Combined with the absorption test, which revealed no significant variation in the total absorbed volume, these results suggested that all samples share a similar final absorption capacity, with different liquid uptake kinetics.

### 3.2. Antimicrobial Properties

**Figure 4:**
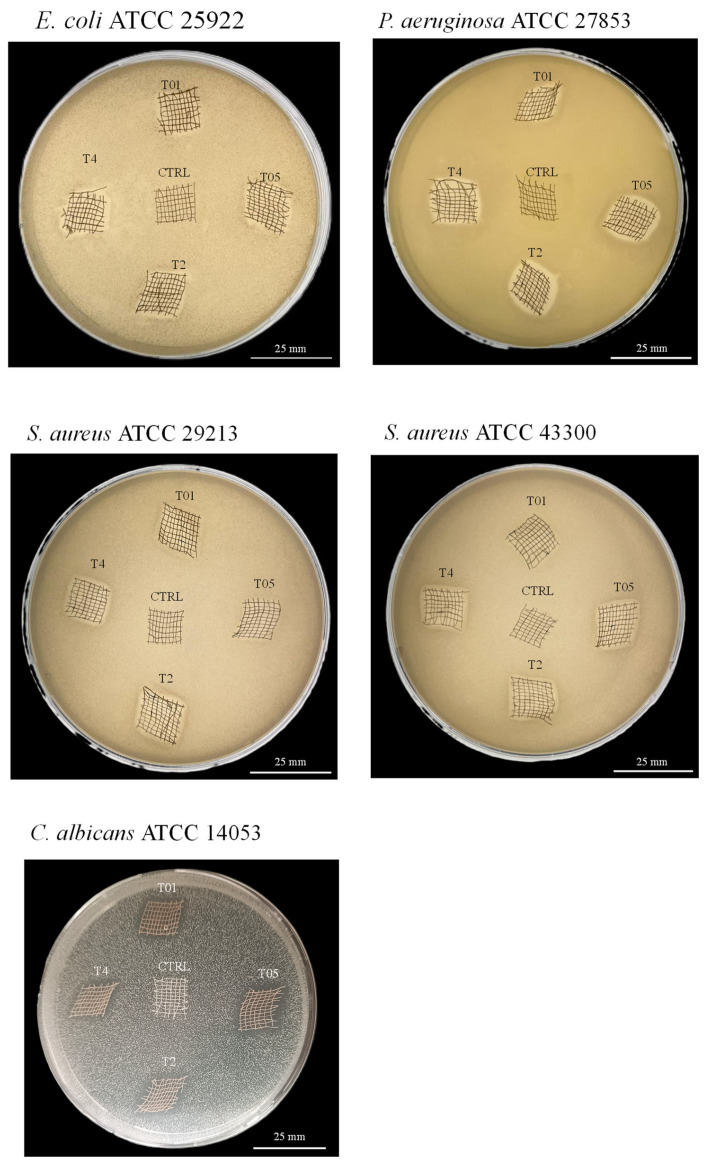
Agar diffusion test on *E. coli* ATCC 25922, *P. aeruginosa* ATCC 27853, *S. aureus* ATCC 29213, *S. aureus* ATCC 43300, and *C. albicans* ATCC 14053. The presence of the zone of bacterial growth inhibition is clearly visible around the samples.

The presence of AgNPs on the surface of cotton gauzes is expected to enhance their antimicrobial properties through both direct contact between silver and the microorganism and the release of Ag^+^ ions. To assess the efficiency of the metal coating, several antimicrobial tests were carried out. Initially, the effect of the treatment was qualitatively evaluated using the agar diffusion method. As shown in Figure 4, the silver-based treatment exhibited greater effectiveness against Gram-negative bacteria (*P. aeruginosa* and *E. coli*), where the antimicrobial effect was already evident for the T01 sample. A similar trend was observed for *C. albicans*. In contrast, for Gram-positive bacteria (*S. aureus* ATCC 29213 and *S. aureus* ATCC 43300), the treatment became effective only at an AgNO_3_ concentration of 0.5%, while T01 showed no significant improvement compared to the control (CTRL). The widths of the inhibition zones are reported in Table 4. For each treated sample, except T01, the width value was higher than 1 mm, confirming ‘good’ antimicrobial efficacy according to the standard ‘SNV 195920-1992’.

**Figure 5:**
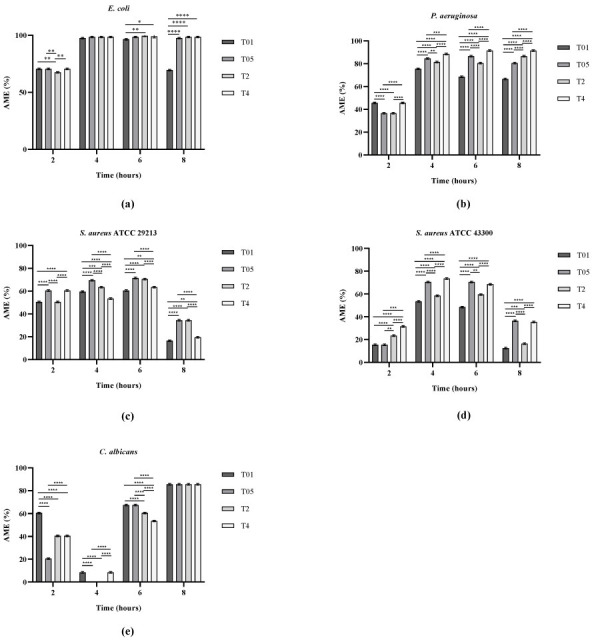
AME (%) for *E. coli* ATCC 25922 (**a**), *P. aeruginosa* ATCC 27853 (**b**), *S. aureus* ATCC 29213 (**c**), *S. aureus* ATCC 43300 (**d**), and *C. albicans* ATCC 14053 (**e**). * *p* < 0.05; ** *p* < 0.01; *** *p* < 0.001; **** *p* < 0.0001.

Moreover, precultured microorganisms with an initial OD_600_ of approximately 0.0005 were incubated in the presence of untreated (CTRL) and treated cotton samples (T01, T05, T2, and T4) to investigate the effect of the coating on microbial growth, through the evaluation of the AME percentage, reported in Figure 5. For Gram-negative bacteria (*E. coli* and *P. aeruginosa*), inhibition of bacterial growth reached maximum values of 98% and 92%, respectively. However, while all treated samples (except T01) exhibited comparable antibacterial efficiency against *E. coli*, *P. aeruginosa* displayed a clear concentration-dependent response, with higher Ag content leading to stronger inhibition. These results are consistent with the qualitative tests, in which the inhibition zone width was similar in all treated samples for *E. coli*, while showing an increase with Ag concentration for *P. aeruginosa*. In the case of Gram-positive bacteria (*S. aureus* ATCC 29213 and *S. aureus* ATCC 43300), the antibacterial effect was less pronounced, with a mean reduction in bacterial growth of approximately 70% at 6 h for both strains, remaining around 40% at longer incubation times, without significant differences among treated samples. These observations are in agreement with the qualitative assays, which revealed smaller inhibition zones compared to Gram-negative bacteria, yet retained antibacterial activity for all samples except T01. Regarding *C. albicans*, due to its slower growth kinetics than bacteria, AME values at early time points (4 h) may appear reduced due to low biomass levels. However, the treatment led to a pronounced antifungal effect, with a maximum growth reduction of 86% after 8 h of incubation. No significant differences were detected among treated samples, aligning with the results obtained from the qualitative assays.

**Figure 6:**
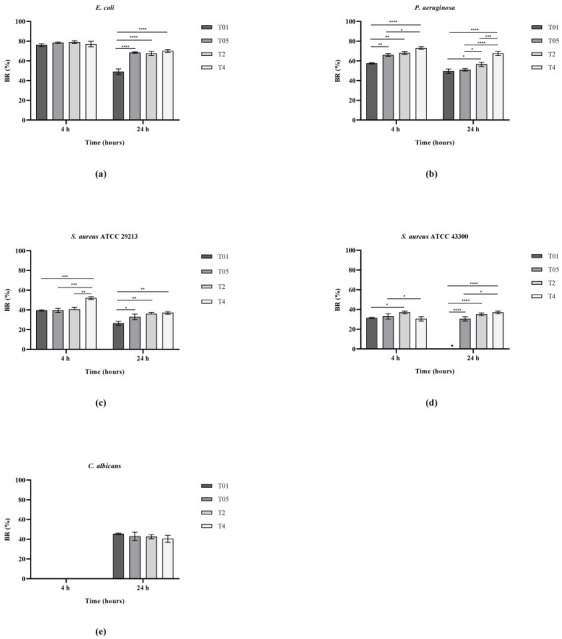
BR (%) for *E. coli* ATCC 25922 (**a**), *P. aeruginosa* ATCC 27853 (**b**), *S. aureus* ATCC 29213 (**c**), *S. aureus* ATCC 43300 (**d**), and *C. albicans* ATCC 14053 (**e**). * *p* < 0.05; ** *p* < 0.01; *** *p* < 0.001; **** *p* < 0.0001.

Finally, the ability of the treated gauzes to inhibit biofilm formation was evaluated using a CV assay after 4 h and 24 h of incubation. As shown in Figure 6, a clear difference was observed between Gram-negative and Gram-positive strains, with higher antibiofilm efficiency against the former in accordance with all other antimicrobial tests. Specifically, for both *E. coli* and *P. aeruginosa*, reductions of approximately 80% and 70% were detected, respectively, with only a slight decrease after 24 h. In contrast, for Gram-positive bacteria, the inhibition was lower, up to about 40%, which remained almost constant between 4 h and 24h. Among the treated samples, T01 exhibited the lowest antibiofilm performance. Regarding *C. albicans*, no detectable biofilm formation was observed at 4 h, likely due to its slower growth rate, whereas after 24 h, in all treated samples, biofilm formation was reduced by approximately 50%, with no significant differences among them.

### 3.3. Biocompatibility Evaluation

**Figure 7:**
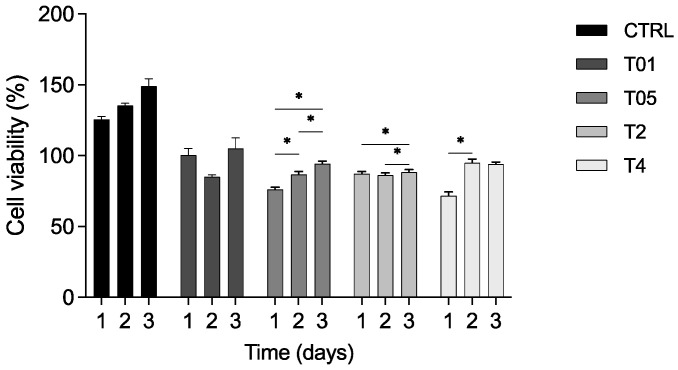
Percentage of cell viability. 3T3 cells were grown in contact with the CTRL, T01, T05, T2, and T4 gauzes at 1, 2, and 3 days, normalized to the TCPS group, as the control. The percentage of cell viability significantly increased from day 1 to day 2, from day 1 to day 3, and from day 2 to day 3 in the T05 group; from day 1 to day 3 and from day 2 to day 3 in the T2 group; from day 1 to day 2 in the T4 group. * *p* < 0.05.

An MTT assay was performed to evaluate if the CTRL, T01, T05, T2, and T4 gauzes influenced the viability of 3T3 cells. The results of cell viability at each time point were expressed as a percentage relative to the TCPS, as the control group. For the CTRL gauzes, cell viability reached 125%, 135%, and 148% after 1, 2, and 3 days, respectively. For the other experimental groups at the same time points, the values were 100%, 85%, and 105% for T01; 76%, 86%, and 94% for T05; 87%, 86%, and 88% for T2; and 72%, 94%, and 94% for T4 (Figure 7). Statistical analysis confirmed a significant increase in viability from day 1 to day 2, day 1 to day 3, and day 2 to day 3 in the T05 group; from day 1 to day 3 and day 2 to day 3 in the T2 group; and from day 1 to day 2 in the T4 group (*p* < 0.05). Nonetheless, the viability values for all samples remained well above the cytotoxicity threshold established by ISO 10993-5:2009 [28], which defines a reduction higher than 30% as indicative of cytotoxicity [26]. Collectively, these results demonstrated that CTRL and treated gauzes are biocompatible.

**Figure 8:**
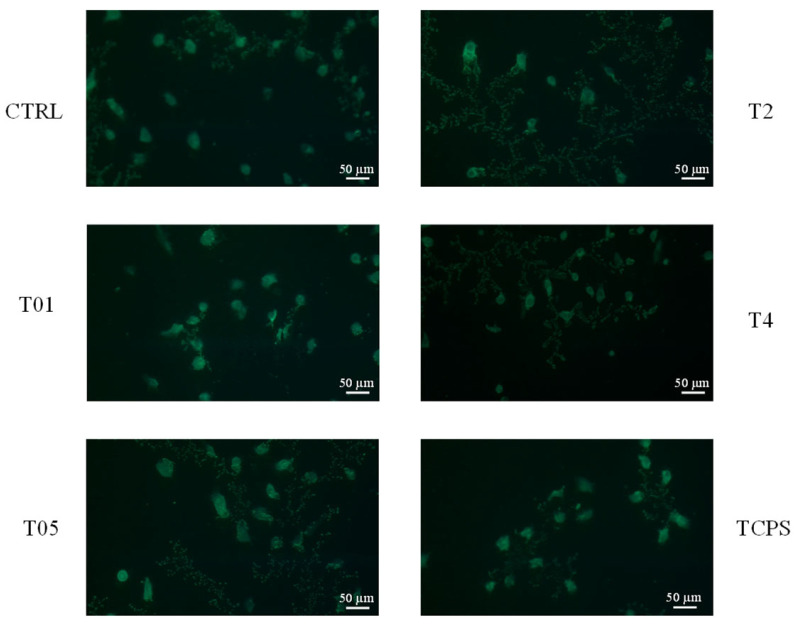
Cell viability analysis of 3T3 fibroblasts using Live/Dead assay. The images show live cells grown in contact with the CTRL, T01, T05, T2, T4 gauzes and on the TCPS as the control at 3 days. Dead cells at each time point were not detected. Scale bar: 50 μm.

The biocompatibility of the gauzes was further examined in vitro through a Live/Dead fluorescence assay conducted on day 3. In this analysis, viable cells were labeled with a green fluorescent marker, while non-viable cells were stained red. At 3 days, fluorescence microscopy images revealed numerous viable 3T3 cells grown in contact with the CTRL, T01, T05, T2, T4 gauzes, showing a distribution similar to that observed in the control samples (TCPS) (Figure 8). No red-stained (dead) cells were detected, thereby confirming the cytocompatibility of the CTRL, T01, T05, T2, and T4 gauzes.

**Figure 9:**
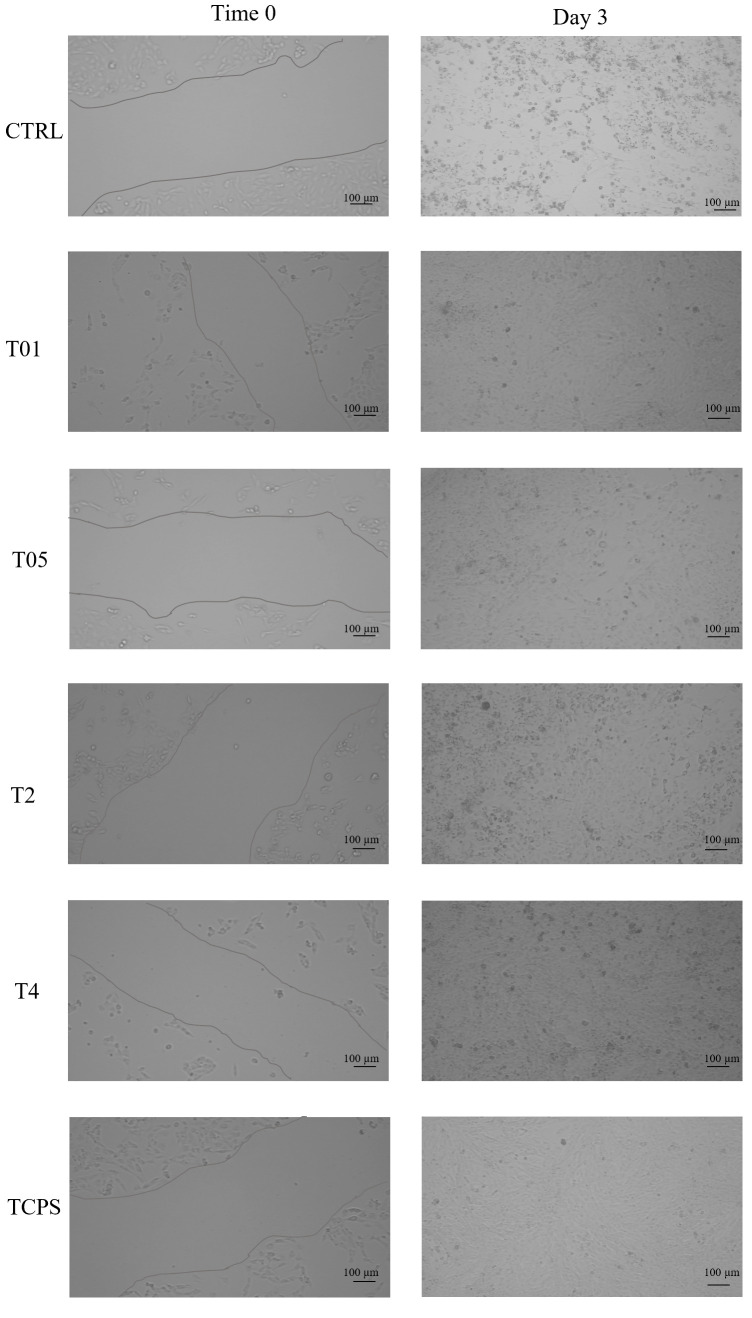
Wound healing analysis through an in vitro scratch assay on 3T3 grown in contact with the CTRL, T01, T05, T2, and T4 gauzes and on the control, at Time 0 and Time 3 days of analysis. At Day 3, the wound closure was completed on the CTRL, T01, T05, T2, T4 gauzes and on the control. Notably, in the CTRL group, fibroblasts repopulated the scratched area less densely compared to the other experimental groups. Magnification: 10×; scale bar 100 μm.

The wound healing ability of the gauzes was evaluated using 3T3 fibroblast cultures exposed to the tested materials and the control. In vitro wound closure was analyzed through a scratch assay, performed at the initial time point (Time 0) and after 3 days of incubation (Day 3). Microscopic observations at Time 0 and Day 3 (Figure 9) displayed the scratched fibroblast monolayers for CTRL, T01, T05, T2, T4, and the TCPS control. After 3 days, complete wound closure (100%) was observed in all experimental groups, including the TCPS control. Although the scratch area was fully closed in every condition, microscopic images revealed that fibroblasts in contact with the silver-treated gauze repopulated the scratched region more densely compared to those grown on the untreated gauze (CTRL), where cell repopulation appeared less compact. These results indicate that the presence of silver may support cell migration and contribute to wound closure, suggesting a potential role of silver-treated gauzes in the wound healing process.

## 4. Discussion

The increasing spread of antibiotic-resistant bacteria represents one of the major challenges in modern medicine, particularly in the prevention and treatment of infections related to wound management. In this context, the modification of traditional healthcare textiles, such as gauzes, with innovative antimicrobial agents has emerged as a promising strategy to reduce the risk of contamination and improve infection control.

The functionalization of gauzes with AgNPs for biomedical applications has been widely discussed in the literature [29,30]. In particular, the dip-coating method followed by UV-assisted in situ photo-reduction, developed by Pollini et al. [31], represents a simple, scalable, and reproducible approach. In this technology, the formation of AgNPs is based on a UV-assisted photo-reduction mechanism where methanol acts not only as solvent but also as a reducing medium. Under UV irradiation, methanol undergoes photochemical activation, leading to the formation of reactive species that promote the reduction of Ag^+^ ions to metallic silver. The synergistic effect of UV exposure and the alcoholic environment enables the in situ formation of silver nanoparticles directly on the cotton fibers, ensuring their homogeneous deposition and strong interaction with the substrate. Previous SEM and EDX analyses have demonstrated the effectiveness of this technology in the development of silver nanocoatings onto textile substrates, also showing their stability and durable broad-spectrum antimicrobial properties for different biomedical applications [32,33]. The aim of this study was to optimize the process parameters for achieving antimicrobial capability specifically for wound healing applications. This optimization study was developed by reducing the content of methanol employed as a reducing agent, replacing the pure solvent with an aqueous solution at 5% *w*/*w*, and testing different concentrations of the AgNO_3_ precursor (0.1% *w*/*w*, 0.5%% *w*/*w*, 2% *w*/*w*, and 4% *w*/*w*) to produce functionalized gauzes (named T01, T05, T2, and T4, respectively) with favorable antimicrobial performance and cytocompatibility.

The first step was to evaluate the amount of unreacted Ag precursor through UV–Vis analyses in both the precursor and washing solutions. The results revealed a progressive increase in unreacted Ag^+^ fractions, particularly in the T2 and T4 samples, where nearly 50% of the precursor was lost during washing and did not contribute to gauze functionalization, mainly due to an incomplete photo-reduction reaction. This result indicated that, for samples treated with percentages of silver precursor higher than 0.5% *w*/*w*, a higher amount of reducing agent is recommended in order to complete the photo-reduction reaction and to avoid the loss of material.

Absorption and retention tests in water, SWF, and blood showed that AgNP treatment did not compromise the hydrophilicity or capillarity of the gauze. Moderate improvements in vertical wicking were observed at the 1 min time point for treated samples, suggesting different liquid uptake kinetics while maintaining a similar final absorption capacity compared to CTRL, a desirable feature for wound dressings, where rapid fluid absorption and distribution support an optimal healing microenvironment [34].

The antimicrobial assays confirmed the effectiveness of AgNP-functionalized gauzes, particularly against Gram-negative bacteria. *E. coli* and *P. aeruginosa* growth were inhibited, with maximum reductions of 98% and 92%, respectively. In contrast, Gram-positive strains (*S. aureus* ATCC 29213 and ATCC 43300) exhibited slightly lower inhibition levels, with a maximum reduction of around 70% at 6 h, decreasing to 40% at longer times, due to bacterial growth over time, while maintaining antibacterial properties. This trend is consistent with previous studies and justified by the different structural characteristics of bacterial cell walls. In fact, Gram-positive bacteria possess a considerably thicker peptidoglycan wall (approximately 30 nm) that provides mechanical protection, whereas Gram-negative bacteria possess a much thinner layer (around 3–4 nm). In addition, the negatively charged peptidoglycan of Gram-positive bacteria can interact with silver ions, reducing their diffusion across the cell envelope and consequently decreasing antimicrobial activity. In contrast, in Gram-negative bacteria the presence of lipopolysaccharides (LPS) enhances membrane interactions and promotes AgNP adhesion via electrostatic forces, thereby amplifying bacterial inhibition even at lower nanoparticle concentrations [35].

The antibiofilm results confirmed the same trend, with stronger inhibition for Gram-negative bacteria. While *S. aureus* biofilms exhibited a reduction of approximately 40%, those of *E. coli* and *P. aeruginosa* were inhibited by 70–80%. In all cases, the antibiofilm activity of AgNPs was lower than their antibacterial efficacy against planktonic cells, in line with previous studies reporting that bacterial biofilms are inherently less susceptible to AgNPs than free-living cells, mainly due to the protective extracellular matrix that encapsulates bacteria and the reduced surface area of aggregated cells exposed to the NPs [36]. The antifungal activity against *C. albicans* was also remarkable, with growth inhibition reaching up to 80% based on OD_600_ measurements and a consistent 50% reduction in biofilm formation across all treated samples after 24 h. This antifungal effect is particularly relevant, as fungal colonization often contributes to delayed wound healing and the development of chronic infections [37].

The cytocompatibility assays confirmed that all AgNP-functionalized gauzes supported 3T3 cell viability, with values remaining well above the ISO 10993-5 cytotoxicity threshold. Live/Dead staining further confirmed the absence of non-viable cells and uniform cell distribution across all samples. The in vitro wound healing potential of treated gauzes, assessed through a scratch assay, showed complete wound closure in all experimental groups. However, fibroblasts grown in contact with silver-functionalized gauzes exhibited denser and more compact repopulation of the scratched area compared to cells on the untreated control gauze. This observation suggests that the presence of silver supports fibroblast viability and may contribute to increased cell density primarily through enhanced cell migration rather than proliferation, as also reported in previous studies [38].

These findings are consistent with previous reports highlighting the beneficial role of silver in accelerating wound healing processes [39,40,41]. A comparison between the present results and those reported in the literature reveals an agreement in the overall trend of antibacterial performance and biocompatibility, despite differences in the methods used for AgNP deposition. In the referenced study, the modified gauze exhibited significantly higher antibacterial and antibiofilm efficacy against Gram-negative bacteria compared to Gram-positive strains [42,43]. A similar behavior was observed in our system, where biofilm inhibition on surfaces reached approximately 80% for Gram-negative bacteria, while a lower inhibition of about 40% was recorded for Gram-positive bacteria. Additionally, while the literature reports acceptable cytocompatibility with cell viability values above 70%, all samples in the present study showed cell viabilities exceeding 80%, confirming high biocompatibility [44].

Overall, these findings demonstrated that AgNP functionalization did not compromise cell compatibility, supporting their suitability for biomedical use. Moreover, a crucial aspect of this study is that, when using very low percentages of reducing agent, increasing the Ag precursor concentration above 0.5% *w*/*w* did not lead to a proportional improvement in antimicrobial performance, suggesting that higher silver loadings are not justified. On the other hand, since the biological outcomes arise from the bioavailable silver released from the treated gauzes, direct quantification of silver release over time would provide additional insight into the correlation between silver availability and biological response. This aspect will be addressed in future work aimed at evaluating the release behavior and long-term performance of the functionalized gauzes under physiologically relevant conditions.

Therefore, in this study, T05 represents the optimal balance between antimicrobial efficacy, biocompatibility, and minimal silver waste, making it the most promising candidate for biomedical applications, with strong potential for clinical translation.

## 5. Conclusions

The aim of this study was to optimize the UV-assisted dip-coating method for the in situ functionalization of cotton gauzes with AgNPs, by reducing the methanol content and identifying the minimum precursor concentration required to achieve effective antimicrobial activity and biocompatibility while minimizing material waste.

Overall, the treatment did not alter the intrinsic properties of cotton gauzes, while providing antibacterial activity against Gram-negative and Gram-positive bacteria, and antifungal effects against *C. albicans*. Furthermore, all treated samples maintained fibroblast viability, confirming their cytocompatibility in accordance with ISO 10993-5 standards, and showed higher wound healing capability compared to the control sample. These results suggested that, when the photo-reducing agent was fixed at 5% *w*/*w*, silver precursor concentrations exceeding 0.5% *w*/*w* led to only marginal improvements in antimicrobial performance, thus highlighting T05 (0.5% *w*/*w* AgNO_3_) as the optimal balance between antimicrobial efficacy, antibiofilm activity, and biocompatibility.

Future work will include wound healing studies to investigate the potential of silver in regenerative medicine, starting from the promising results achieved in this study and those reported in the literature. Furthermore, coupling AgNPs with biocompatible polymers or natural agents will be explored to achieve sustained silver release in the development of more advanced devices for wound healing applications.

## Figures and Tables

**Table 1:** Unreacted and reacted [Ag^+^] per sample.

Sample	Unreacted [Ag^+^] (ppm)	Reacted [Ag^+^] (ppm)	Fraction Unreacted/Reacted
T01	96	251	28/72
T05	124	283	30/70
T2	793	423	65/35
T4	548	496	53/47

**Table 2:** Average retention times of samples with different fluids.

Sample	Mean Time of Drying (min)		
	Water	SWF	Blood
CTRL	45	90	75
T01	60	90	90
T05	60	90	105
T2	60	90	75
T4	60	90	75

**Table 3:** Vertical wicking ability of samples at different times.

Sample	Vertical Wicking Measurements (cm)		
	1 min	5 min	10 min
CTRL	1.77 ± 0.15	3.17 ± 0.15	3.63 ± 0.21
T01	2.40 ± 0.10 **	3.40 ± 0.36	4.10 ± 0.26 *
T05	2.23 ± 0.21 *	3.17 ± 0.15	4.00 ± 0.17
T2	2.23 ± 0.21 *	3.00 ± 0.06	3.67 ± 0.15
T4	2.10 ± 0.17	3.17 ± 0.15	3.67 ± 0.15

* *p* < 0.05; ** *p* < 0.01 (vs. CTRL).

**Table 4:** Width of the inhibition zone in agar diffusion tests.

Sample	Dimension of the Inhibition Zone (mm)				
	*E. coli*	*P. aeruginosa*	*S. aureus* ATCC 29213	*S. aureus* ATCC 43300	*C. albicans*
CTRL	/	/	/	/	/
T01	1.19 ± 0.06	0.80 ± 0.25	/	/	1.12 ± 0.13
T05	1.10 ± 0.18	1.43 ± 0.12	0.67 ± 0.08	1.03 ± 0.07	1.18 ± 0.08
T2	1.03 ± 0.35	1.73 ± 0.08	1.09 ± 0.19	1.16 ± 0.26	1.20 ± 0.15
T4	1.22 ± 0.14	1.38 ± 0.11	1.53 ± 0.02	1.45 ± 0.40	1.23 ± 0.04

## Data Availability

The original contributions presented in this study are included in the article. Further inquiries can be directed to the corresponding author.

## Abbreviations

The following abbreviations are used in this manuscript:

AgNPs	Silver Nanoparticles
ROS	Reactive Oxygen Species
MW	Molecular Weight
PBS	Phosphate-Buffered Saline
UV	Ultraviolet
RT	Room Temperature
SWF	Simulated Wound Fluid
CV	Crystal Violet
MRSA	Methicillin-Resistant
TSB	Tryptic Soy Broth
PDA	Potato Dextrose Agar
OD	Optical Density
ATCC	American Type Culture Collection
AME	Antimicrobial Efficacy
CFUs	Colony-Forming Units
BR	Biofilm Reduction
DMEM	Dulbecco’s Modified Eagle Medium
FBS	Fetal Bovine Serum
TCPS	Tissue Culture Polystyrene
DMSO	Dimethyl Sulfoxide
WC	Wound Closure
SD	Standard Deviation
